# TRAINING, SUPPORTING, AND ENGAGING VOLUNTEERS 55+ IN VIRTUAL COMPANIONSHIP FOR PEOPLE WITH DEMENTIA

**DOI:** 10.1093/geroni/igae098.3085

**Published:** 2024-12-31

**Authors:** Jessica Herpst, Quincy Samus, Katherine Marx, Kathleen Kirchner

**Affiliations:** Alzheimer’s Association - Greater Maryland Chapter, Baltimore, Maryland, United States; Johns Hopkins University, Baltimore, Maryland, United States; Johns Hopkins University, Baltimore, Maryland, United States; Johns Hopkins University, Baltimore, Maryland, United States

## Abstract

Volunteers play an essential role in our communities and societal health. Engaging, training, and mobilizing a critical mass of volunteers to address social isolation and other dementia care needs may be a potent approach to address the dementia care crisis, and one that is attractive to older volunteers seeking to remain in impactful post-retirement roles. A collaboration between Johns Hopkins University and the Alzheimer’s Association evaluated the feasibility and acceptability of a virtual companionship program for community-residing people with dementia (PWD) delivered by trained Volunteers 55 years and older in the MEMORI Corps trial. We aim to describe the feasibility and lessons learned for recruitment, training, and support of the program volunteers. 52 eligible volunteers were recruited and 35 completed initial volunteer onboarding and training modules and were matched to provide regular virtual visits to a person living with dementia for a 12-week intervention. An approachable and responsive training program and learning environment was created that included didactic learning modules as well as initial and experiential learning opportunities supported by an interdisciplinary clinical team and weekly group-based training support. Common implementation challenges included volunteer long term commitment, difficulty using technology or low confidence using Zoom, scheduling of sessions, maintaining professional boundaries, and addressing dementia symptoms during sessions. Perceived benefits included camaraderie among volunteers; building confidence in using technology, in working with PWD and interpersonal skills; learning about healthy aging; and feeling more productive and connected to the community. Overall, volunteer engagement was feasible for volunteers and had perceived benefits.

